# Already!

**Published:** 1892-03-26

**Authors:** 


### PASSING TOPICS.

### ALREADY!

In a recent issue the *Times* announced that a Bill introduced by Mr. Mather, M.P., designs to confer upon the more important local bodies, namely, County Councils, Borough Councils, and Urban Sanitary Authorities, the power of contributing to the support of those hospitals, infirmaries, &c., which are maintained by voluntary subscriptions—of course with the inevitable condition, among others, that when a subscription is made, the local authority shall be duly represented upon the governing body of the institution.

We shall be interested to see what amount of support Mr. Mather is able to command for an effort doubtless made with the very best of intentions, but which will be viewed with repugnance by those whom it most concerns. The position he takes up is reached logically enough, when we witness the straits to which many of our hospitals are reduced by want of money, but none the less we may question whether the House of Commons is as yet prepared to take action, the result of which must be to convert voluntary into rate-supported institutions.

It is perfectly true that the public have only themselves to blame if any such calamity impend, and whatever the immediate prospect, the mere fact that a proposal of the kind is possible, is sufficient to raise an uncomfortable suspicion that danger is ahead. At the very time Mr. Mather is proposing to bring in his Bill, we have Lord Sandhurst protesting that his chief object in moving for the Select Committee, over which he presides, was chiefly due to his anxiety to keep the hospitals off the rates, and whatever our opinion as to the way the Lords' inquiry has been conducted, we think Parliamentary action should certainly have been postponed until the Committee had reported.

Be the fate of Mr. Mather's Bill, however, what it may, it behoves the voluntary hospitals to regard its introduction as one more sign of the times. They will be wise to weigh it with all the attendant circumstances thrown into the scale, and better they should attach too much rather than too little importance to its occurrence. The affairs of our hospitals have lately been rather more in evidence than some of them have liked, but for our part we see no good in shirking the issues involved in the admission that they are always hard put to it for the means to exist, and that the voluntary system has not yet proved itself capable of providing for all necessities. Their authorities must be prepared for all sorts of proposals, and it is obvious that when we insist, and rightly, upon the duty which every member of the community owes to these indispensable institutions, we put an argument into the mouths of those to whom local boards, county councils and such like, constitute a panacea for every evil.

It needs not only an intimate acquaintance with hospital daily life, but a nice perception of the somewhat impalpable though powerful evidences of the value of a benevolent administration, to enable a man to distinguish readily between the ways of the voluntary and the rate-supported institution. All the more reason that those who are capable of appreciating the advantages the voluntary hospital possesses should bestir themselves to ensure that it shall be by no fault of theirs, if its benefits are lost to the next generation.

Agricola.

